# Design, Optimization and Characterization of a Transfersomal Gel Using Miconazole Nitrate for the Treatment of Candida Skin Infections

**DOI:** 10.3390/pharmaceutics10010026

**Published:** 2018-02-23

**Authors:** Mona Qushawy, Ali Nasr, Mohammed Abd-Alhaseeb, Shady Swidan

**Affiliations:** 1 Department of Pharmaceutics, Faculty of Pharmacy, Sinai University, Alarish, North Sinai 45511, Egypt; 2 Department of Pharmaceutics, Faculty of Pharmacy, University of Tabuk, Tabuk 471, Saudi Arabia; 3 Department of Pharmaceutics, Faculty of Pharmacy, Port Said University, Port Said 42511, Egypt; 4 Department of Pharmacology and Toxicology, Faculty of Pharmacy, Damanhour University, Damanhour 22511, Egypt; m.abdelhasseb@su.edu.eg; 5 Department of Pharmaceutics, Faculty of Pharmacy, The British University in Egypt, Cairo 11837, Egypt

**Keywords:** Transfersomes, miconazole nitrate, antifungal activity, entrapment efficiency, Carbapol 934

## Abstract

Miconazole nitrate (MIC) is an antifungal drug used for treatment of superficial fungal infections. However, it has low skin permeability. Hence, the objective of this study was to prepare miconazole nitrate using Transfersomes to overcome the barrier function of the skin. MIC Transfersomes were prepared using a thin lipid film hydration technique. The prepared Transfersomes were evaluated with respect to entrapment efficiency (EE%), particle size, and quantity of in vitro drug released to obtain an optimized formulation. The optimized formulation of MIC Transfersomes was incorporated into a Carbapol 934 gel base which was evaluated in comparison with a marketed product (Daktarin® cream 2%) for drug content, pH, spreadability, viscosity, in vitro permeation, and in vitro and in vivo antifungal activity. The prepared MIC Transfersomes had a high EE% ranging from (67.98 ± 0.66%) to (91.47 ± 1.85%), with small particle sizes ranging from (63.5 ± 0.604 nm) to (84.5 ± 0.684 nm). The in vitro release study suggested that there was an inverse relationship between EE% and in vitro release. The kinetic analysis of all release profiles was found to follow Higuchi’s diffusion model. All independent variables had a significant effect on the dependent variables (*p*-values < 0.05). The prepared MIC transfersomal gel showed higher antifungal activity than Daktarin® cream 2%. Therefore, miconazole nitrate in the form of Transfersomes has the ability to penetrate the skin, overcoming the stratum corneum barrier.

## 1. Introduction

Fungal infections are superficial infections which occur in the skin, nails, and mucous membranes. Candidiasis is one of the most widespread types of superficial fungal infections, and can invade into deep tissue in cases of weakness in the immune system. It usually affects wet, warm, and furrowed areas such as the underarms and intergluteal areas [1].

Topical treatment of fungal infections is usually preferred as opposed to systemic treatment, as the drug is delivered directly to the infected site, with decreased side effects and improved patient compliance [2]. However the stratum corneum, which is the outermost layer of the skin, represents the main barrier for drug penetration. Hence it is necessary to design a drug delivery system for antifungal drugs which has the ability to overcome the barrier properties of the stratum corneum [2,3].

Miconazole nitrate (MIC) is a wide-spectrum anti-fungal drug that has an imidazole group and is used for the treatment of candidiasis. The systemic efficacy of MIC is low due to poor water solubility and intensive hepatic transformation [4]. The mechanism of action of MIC is based on the inhibition of ergosterol biosynthesis (resulting in fungal cell membrane lysis) and peroxidase inhibition, which leads to accumulation of peroxide within the cell, resulting in cell death [1,2].

The topical application of MIC is problematic in the treatment of cutaneous diseases due to poor skin penetration. Conventional formulations are given in higher doses to overcome this issue and compensate for low permeability. In recent years, the use of lipid vesicles as carriers for topical drugs has attracted great attention due to their ability to overcome the barrier properties of the skin [5].

Transfersomes are ultra-flexible vesicles with a bilayer structure. They can penetrate the skin easily and overcome the barrier function by squeezing through the intracellular lipid of the stratum corneum [6]. After application of Transfersomes on the skin, they move from the dry stratum corneum to a deep hydrated layer according to the osmotic gradient. The presence of surfactant in their structure helps in solubilizing the lipid in stratum corneum and permits high penetration of the vesicles [5].

The aim of this study was to prepare and evaluate MIC transfersomal gel to enhance skin penetration and increase antifungal activity. Candidiasis is used as a model disease to evaluate the antifungal activity of the prepared MIC transfersomal gel in comparison with a marketed product (Daktarin® cream 2%).

## 2. Experimental Section

### 2.1. Materials

Miconazole nitrate was a gift from Medical Union Pharmaceuticals Company, Ismailia, Egypt. Daktarin^®^ cream was purchased from Janssen/Mina Pharm International Pharmaceutical Industries Company, Cairo, Egypt. Soya lecithin was provided by the Lipoid International Pharmaceutical Industries Company, Ludwigshafen, Germany. Span 80 and Tween 80 were obtained from the Sigma Chemical Company, Taufkirchen, Germany. Potassium dihydrogen phosphate and sodium hydroxide were obtained from Pure Lab, USA. Methanol and chloroform were purchased from El-Nasr Pharmaceutical Chemical Company, Cairo, Egypt.

### 2.2. Methods

#### 2.2.1. Preparation of MIC-Loaded Transfersomes

MIC Transfersomes were prepared using the thin lipid film hydration technique [3] with three independent factors including the type of surfactant (X1), total lipids (X2), and the phospholipid to surfactant ratio (X3), as shown in Table 1. A factorial design (2^3^) was applied to prepare eight different formulations of MIC Transfersomes. Precise amounts of phospholipids, surfactant (edge activator), and drug were dissolved in a mixture of organic solvents consisting of chloroform and methanol (2:1, v/v) in a dry, round-bottom flask. The organic solvent was allowed to evaporate using a Heidolph rotavap (P/N Hei-AP Precision ML/G3, Schwabach, Germany) adjusted to 60 rpm, at 45 °C under low pressure to prepare a thin lipid film on the wall of the round-bottom flask. The final residuals of the solvents were allowed to be removed under vacuum overnight. The dry thin lipid film was subjected to hydration with phosphate-buffered saline (pH 7.4) by rotation for 1 h at 60 rpm at room temperature. The formed lipid vesicles were allowed to swell for 2 h at room temperature (25 °C). The multilamellar lipid vesicles (MLVs) were then sonicated using the Sonicator Digital Sonifier (Branson, Danbury, CT, USA) for 15 min to reduce the vesicle size and stored at 4 °C for further investigation [7].

#### 2.2.2. Characterization of Transfersomes

##### Determination of Entrapment Efficiency (EE %)

One milliliter of MIC Transfersomes suspension was centrifuged at 15.000 rpm for 1 h to allow the separation the entrapped drug from the un-entrapped drug. After removal of the supernatant, the sediment was lysed using methanol and then analyzed spectrophotometrically at 272 nm using a UV spectrophotometer, (Shimadzu, Kyoto, Japan). The EE% of MIC in the prepared Transfersomes was calculated applying the following equation [8]:

EE% = [Amount of Entrapped MIC/Total Amount of MIC] × 100

##### Determination of Particle Size, Zeta Potential and Polydispersity Index

Particle size, zeta potential and polydispersity index were measured for all prepared MIC Transfersomes using the dynamic light scattering (DLS) technique at 25 °C using the Particle Size System, (Zetasizer, Malvern Instruments Ltd., Malvern, UK). The MIC-loaded transfersomal colloidal dispersion was diluted with purified water before being subjected for measurements [9].

##### Determination of In-Vitro Skin Permeation of MIC Transfersomes

The in vitro skin permeation of MIC Transfersomes was done using Franz’s diffusion cell apparatus (Maharashtra, Mumbai, India) (area = 1.7 cm^2^). The rat skin was fixed between the donor and receptor compartments of the diffusion cell. The rat skin was adjusted with the stratum corneum layer facing into the donor compartment, while the dermis faced the receptor compartment [2]. The receiver phase was comprised of 30 mL of methanolic phosphate buffer saline pH 7.4 (1:1 v/v). The dissolution medium was stirred at a speed of 100 rpm, and the temperature was kept constant at 37 °C ± 0.5 °C for 24 h. The MIC Transfersomes suspension, equivalent to 5000 μg of drug, was placed in the donor compartment and samples of dissolution medium (2 mL) were collected at different time intervals (1, 2, 4, 6, 8, 10, 12 and 24 h). The withdrawn samples were replaced with an equal volume of fresh dissolution medium. The experiment was done in triplicate and with measurement of means and standard deviations. The amount of MIC permeated was determined by spectrophotometric analysis at a wavelength of 272 nm against methanolic phosphate buffer saline pH 7.4 (1:1 v/v) as a blank [1]. The cumulative amount of permeated MIC was plotted against time and the MIC flux at steady state was determined from the slope of the straight line. The results of MIC permeation were kinetically treated to determine the best order of drug permeation [10].

##### Optimization of the Formulation Ingredients

Optimization of formulation ingredient was done in order to determine the values of X1, X2, and X3, which required getting an optimized formula with optimum values of Y1, Y2 ,and Y3 using Statgraphics (R) plus (version 4, Manugistics Inc., Rockville, MD, USA) [11].

##### Morphology of Optimized MIC TRANSFEROSOME Formula

The morphology of optimized formula was determined using a transmission electron microscope (JTEM model 1010, JEOL®, Tokyo, Japan). This determination was performed by applying one drop of optimized transfersomal suspension to a collodion-coated copper grid, which was then left for about 2 min to allow drying and adherence of the Transfersomes to the collodion. Subsequently, one drop of uranyl acetate solution was added for 1 min on a grid. The sample was dried, followed by examination with TEM [12].

##### Fourier Transforms Infrared Spectroscopy

Infra red (IR) spectroscopy was used to ensure the compatibility between the drug and the Transfersomes component [8]. The IR spectra of MIC, a physical mixture with Span 80, a physical mixture with Tween 80, MIC Transfersomes prepared with Span 80, and MIC Transfersomes prepared with Tween 80 were obtained by using a Fourier-transform infrared spectrophotometer. Each sample was mixed with potassium bromide, compressed into a disc, and then scanned from 4000 cm^−1^ to 400 cm^−1^ [10].

##### Differential Scanning Calorimetry (DSC)

Thermal analysis of drug and additives was performed with a differential scanning calorimeter (Shimadzu, Kyoto, Japan). DSC analysis was done for the pure MIC, a physical mixture with Span 80, a physical mixture with Tween 80, MIC Transfersomes prepared with Span 80, and MIC Transfersomes prepared with Tween 80. The DSC thermograms were obtained at temperature ranging from 0 to 250 °C and a scanning rate of 10 °C/min [7].

#### 2.2.3. Preparation of MIC Transfersomal Gel

A 2% MIC transfersomal gel was formulated using 1% Carbapol 934 as a gelling agent. The accurate weight of polymer was sprinkled into a beaker containing 60 mL boiling distilled water, and then soaking was allowed overnight. The transfersomal dispersion containing 2% MIC was added with continuous stirring to allow homogeneous distribution of MIC Transfersomes within the gel base [13]. Methylparaben and propyleparaben were used as preservatives [14]. The dispersion was neutralized by addition of sodium hydroxide dropwise, with continuous mixing until a homogenous gel was obtained. Then the amount of added base was controlled to adjust the pH of prepared gel to pH 6.5 using a pH meter, and the total weight was adjusted to 100 g using distilled water.

#### 2.2.4. Evaluation of MIC Transfersomal Gel

The prepared MIC transfersomal gel was evaluated in comparison with Daktarin® cream 2% for homogeneity, spreadability, pH, drug content, viscosity, in vitro permeation, and antifungal activity [14].

##### Homogeneity

It is important for patient compliance to determine the homogeneity of semisolid dosage forms which are applied topically on the skin. This was done by pressing small quantity of both gels (MIC transfersomal gel and Daktarin® 2%) between the thumb and the index finger. The consistency was determined as homogeneous or not [15].

##### Spreadability

The spreadability of both MIC transfersomal gel and Daktarin® 2% determined by pressing 0.5 g of each between two transparent circular glass slides. Maximum spreading was allowed by leaving them for 5 min. [1]. The diameter of the formed circle was measured to express the spreadability.

##### pH Measurement

One gram of each type of gel was dispersed in 20 mL of distilled water, and a digital pH meter (JENWAY, Staffordshire, UK) was used to determine the pH value. The measurement was performed three times and the mean ± SD was calculated [15].

##### Drug Content Determination

The MIC content was measured by placing 0.5 g of each gel (equivalent to 10 mg of drug) onto a clean volumetric flask (100 mL) and completing the volume with methanol. This was then stirred for 2 h and allowed to stand for 24 h. The solution was filtered and samples were analyzed spectrophotometrically at 272 nm [15].

##### Viscosity Measurement

The viscosity of both gels was measured by a Brookfield viscometer R/S+RHEOMETER, (Brorokfield Inc., MA, USA) using spindle number CC 14 rotated at a speed of 5 rpm for a 10-s run time at 37 °C [1].

##### In-Vitro Skin Permeation

This test was done for the MIC transfersomal gel and Daktarin® cream 2% using a cell diffusion apparatus as mentioned before, and the results were analyzed by a paired T-test using SPSS software (Version 19). The results of MIC permeation were kinetically treated to determine the order of drug permeation. The steady state flux (J_SS_) was calculated from the slope of the linear part of the cumulative amount of MIC permeated per unit area (μg/cm^2^) against a time (h) plot [7]. The permeability coefficient (KP) of MIC through the rat skin was calculated according to the following equations [3]:

KP = J_SS_/C_o_

where C_o_ is the initial MIC concentration.

##### The In-Vitro Antifungal Activity

Antifungal activity was determined using the cup plate technique. The marketed product (Daktarin® cream 2%) was used as a standard. The standard and MIC transfersomal gel (test) was taken into cups bored into sterile sabouraud dextrose agar previously seeded with *Candida albicans*. The plate was incubated for 48 h at 25 °C after allowing diffusion of formulation for 2 h. The zones of inhibition were measured in mm after 48 h for the test and standard [15,16].

##### In-Vivo Antifungal Evaluation of MIC Transfersomal Gel

The in vivo experiment was carried out according to the guidelines after approval of the ethics committee of Faculty of Pharmacy at Damanhour University (approval number 1217PT3).

#### 2.2.5. Preparation of Immunosuppressed Animals

Albino rats (150–180 g) were obtained from the modern veterinary office for laboratory animals (Cairo, Egypt). All animals were allowed to acclimatize under standard animal house conditions for one week before assignment to the experimental protocol. Immunosuppression was performed to produce a heavy cutaneous infection in order to evaluate the effect of the MIC transfersomal gel in the treatment of deep fungal infection in comparison to Daktarin® cream 2%. Rats were immunosuppressed due to administration of intravenous methylprednisolone (5 mg/kg) [16] for 3 days, and then fungal infection was induced [17].

#### 2.2.6. Preparation of the Fungal Strain

*Candida albicans* was allowed to grow on sabouraud dextrose agar for period of 48 h at 30 °C. Subsequently, the cells were collected, washed, and suspended in sterile saline to obtain a final concentration of 10^7^ CFU/mL [17].

#### 2.2.7. Induction of Fungal Infection (*Candida albicans*)

All rats were prepared by shaving about 4 cm^2^ of hair. Each rat was injected intradermally with 100 μL of 10^7^ CFU/mL *Candida albicans* suspension in the middle part of shaved area. The injected area was rubbed until the slight edema disappeared. The fungal infection was observed in the affected area after 72 h [17].

#### 2.2.8. Experimental Design

Rats were divided to four groups of six animals each. Group 1 served as the negative control without fungal infection. After induction of fungal infection for 72 h, Group 2 was kept as positive control. Groups 3 and 4 received treatment through the application of MIC transfersomal gel and Daktarin® cream 2%, respectively, topically for 10 days. During the treatment period, clinical and post mortem examinations were performed.

#### 2.2.9. Clinical Investigations

All rats were kept under observation before and during experiments in order to identify any clinical lineaments related to fungal infection. Symptoms such as rashes, white substances over affected areas, red or purple patches, cracking, scaling, maceration, erythema, and pimples filled with puss were observed and recorded.

#### 2.2.10. Histopathological Examination

At the end of the experiments, rats were anesthetized using slight ether and then sacrificed. Skin of the affected area was removed, fixed with 10% formaldehyde, and then paraffin-blocked. Slides were prepared from the paraffin blocks and stained with haematoxylin–eosin dyes. Examination of the skin samples was done using a microscope to characterize inflammation symptoms, as well as epidermal and dermal changes [17]. A scoring system was used to quantify the fungal infection as reported by Satyam et al. [18].

## 3. Results and Discussion

### 3.1. Preparation of MIC Transfersomes

Three different independent variables were used which include: type of surfactant (X1), total lipids (X2), and phospholipid-surfactant ratio (X3); see Table 1. The independent variables were screened using a multilevel factorial design (2^3^) and eight different formulations of MIC Transfersomes were obtained, as represented in Table 2. All formulations were prepared using the thin lipid film hydration technique and then evaluated for entrapment efficiency, particle size, and transdermal flux to determine the optimized formula [11,19].

### 3.2. Entrapment Efficiency of MIC in Prepared Transfersomes (Y1)

**Figure 1:**
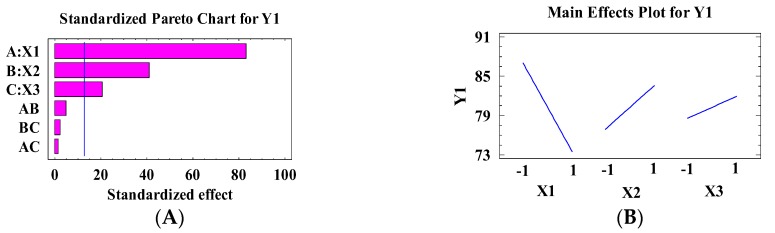

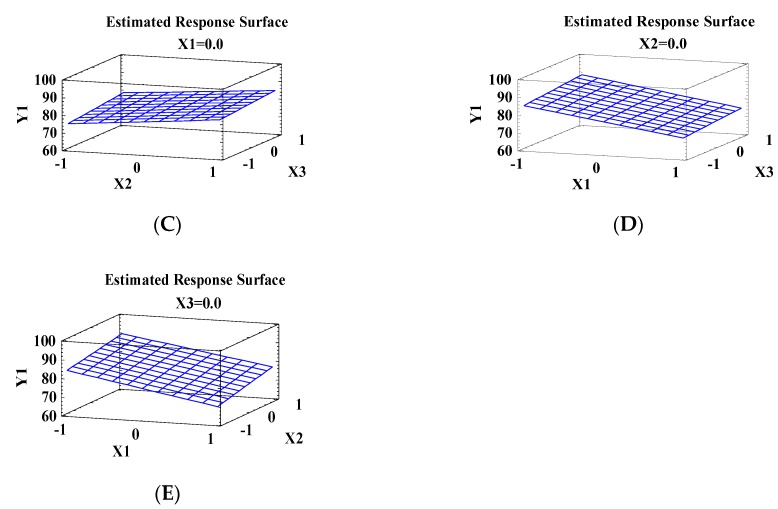
The effect of independent variables on the EE% (Y1) including the Pareto chart (**A**), main plot effect (**B**), and three-dimensional contour plots (**C**–**E**).

As shown in Table 3, it was found that the prepared MIC Transfersomes exhibited a good EE%, with values ranging from (67.98 ± 0.66%) for F6 to (91.47 ± 1.85%) for F1. Figure 1 illustrates the effect of X1, X2, and X3 on the EE% of MIC using statgraphic plus software. As shown on the Pareto chart (Figure 1A), XI, X2, and X3 have significant effects on the entrapment efficiency, with *p*-values of 0.0076, 0.0154, and 0.0306 respectively (see Table 4). The linear regression models for the EE% of MIC Transfersomes are represented in Equation (1) as obtained from a multilevel factorial design study.

Y1 = 80.2088 − 6.77875 × X1 + 3.35625 × X2 + 1.69125 × X3 + 0.41375 × X1 × X2 − 0.14125 × X1 × X3 − 0.21125 − X2 − X3
(1)

The main effect plot for the EE% (Figure 1B) showed that the EE% of MIC in prepared Transfersomes decreased with increasing X1, and increased as X2 and X3 increased. The same results were obtained through estimated response surface (Figure 1C–E) which illustrates the effect of two variables on EE% when the third one is kept at an intermediate value.

From the results of EE%, it was found that Transfersomes prepared with Span 80 have a higher EE% than those prepared with Tween 80. These results may be attributed to the hydrophilic lipophilic balance (HLB) values of these surfactants. The HLB values of Span 80 and Tween 80 were 4.3 and 15, respectively. Hence, according to HLB values, the affinity of surfactant to phospholipid was expected to be higher in case of Span 80 than in case of Tween 80 due to the higher lipophilicity of Span 80 [2,7].

The EE% was increased in the case of 600 mg of total lipids and a phospholipid–surfactant ratio of 90:10, as compared to 300 mg of total lipids and a phospholipid–surfactant ratio of 80:20, respectively. These results could be explained by the increase in the rigidity of the lipid bilayer structure [12] and the increase in the ratio of lipid volume as compared to the interior aqueous volume in the prepared vesicles. These results are in good agreement with those of Abdallah et al. who reported that the EE% of nystatin in the prepared Transfersomes was higher in case of Span 80 than in case of Tween 80 [7].

### 3.3. Vesicle Size (Y2), Zeta Potential, and Polydispersity Index of Prepared MIC Transfersomes

The prepared MIC Transfersomes were tested for particle size, zeta potential, and polydispersity index, as shown in Table 3. The vesicle size ranged from (63.5 ± 0.604 nm) for F1 to (84.5 ± 0.684 nm) for F6. From the results of particle size, it was found that all prepared MIC Transfersomes have a particle size less than 200 nm, and as such are effective for transdermal applications [20].

The linear regression models for particle size of MIC Transfersomes are represented in Equation (2) as obtained from multilevel factorial design study.

Y2 = 74.1875 + 4.7625 × X1 − 4.2375 × X2 − 1.4125 × X3 − 0.5625 × X1× X2 + 0.3625 × X1 × X3 − 0.3875 × X2 × X3
(2)

**Figure 2:**
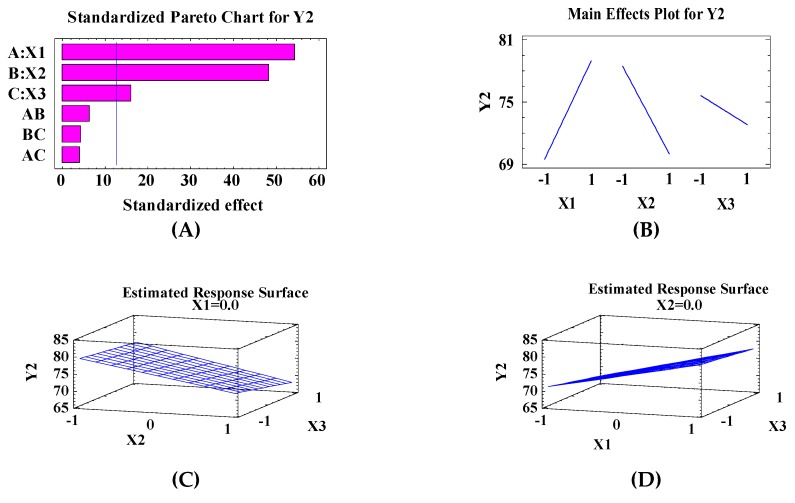

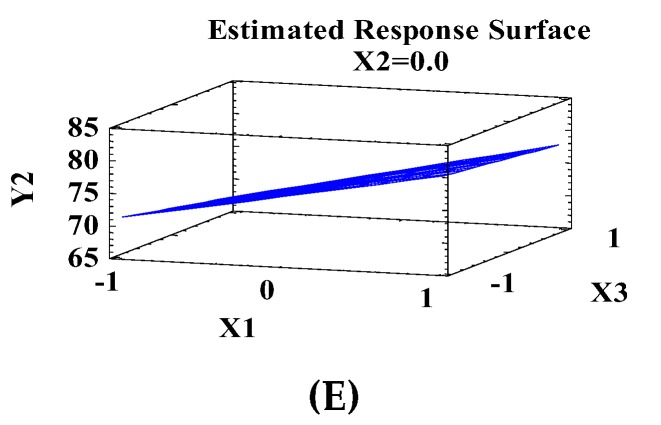
The effect of independent variables on the vesicle size (Y2) including the Pareto chart (**A**), main plot effect (**B**), and three-dimensional contour plots (**C**–**E**).

Figure 2 illustrates the effect of the different independent variables (X1, X2, and X3) on the vesicle size of MIC Transfersomes using the Statgraphic Plus program. As shown in Figure 2A the Pareto chart illustrated that X1, X2, and X3 have significant effects on the vesicle size, with *p*-values of 0.0117, 0.0131, and 0.0394, respectively (see Table 4).

The main effect plot and estimated response surface (which distinguish the effect of two variables on vesicle size when the third one kept in the intermediate value) showed that the vesicle size of the prepared MIC Transfersomes increased with increasing X1, and decreased as X2 and X3 increased. See Figure 2B–E.

From the previous results it was found that MIC Transfersomes prepared with Span 80 have a smaller vesicle size than those prepared with Tween 80. This may be attributed to the general concept of the use of surfactant with a lower HLB which resulted in the preparation of vesicles with smaller sizes [21]. Hence, the Transfersomes prepared with Span 80 (lipophilic surfactant) have the smaller size than those prepared with Tween 80 (a hydrophilic surfactant). The direct proportionality between vesicle size and surfactant HLB can be explained by the reduction in surface free energy resulting from high hydrophobicity. The same explanation applies for the decrease in vesicle size as the total lipids increased.

It was also found that vesicle size was reduced when increasing the phospholipid–surfactant ratio from 80:20 to 90:10. This may be due to the decrease in the amount of surfactant, which leads to incomplete maturation of vesicles and thus a reduction in their sizes [3].

As shown in Table 3, all formulations have a positive zeta potential ranging from (+16.98 mv) for F1 to (+35.74 mv) for F4. The positive values of zeta potential of MIC-loaded Transfersomes are mainly due to the presence of cationic nitrogen atoms in the structure of MIC nitrate (which is an ionic drug). This positive charge of the drug dominates over the lipids and the neutral charge of the non-ionic surfactants used. The magnitude of zeta potential in all prepared formulations is sufficiently high to prevent coagulation and provide stability for the vesicles. These findings were supported by Mahmood et al., who prepared raloxifene nano-Transfersomes and found that the prepared vesicles had positive zeta potential values [22]. The positive zeta potential of vesicles containing MIC nitrate as drug was in accordance with the results of Shah et al., who prepared free and MIC-loaded solid lipid nanoparticles and found that the former were negatively charged. However, after MIC loading the zeta potential became highly positive [23].

The size distribution is another parameter of high importance which is expressed by a dimensionless value called the polydispersity index (PDI). This parameter is a measure of particle homogeneity and it varies from 0.0 to 1.0. The closer to zero the PDI value is, the more homogenous the vesicles are. As represented in Table 3, the values of the polydispersity index for all formulations were less than 0.5, indicating a narrow and homogenous size and distribution. This in turn indicates more uniform vesicles with higher physical stability [24].

### 3.4. In Vitro Permeation of MIC from Prepared Transfersomes (Y3)

**Figure 3:**
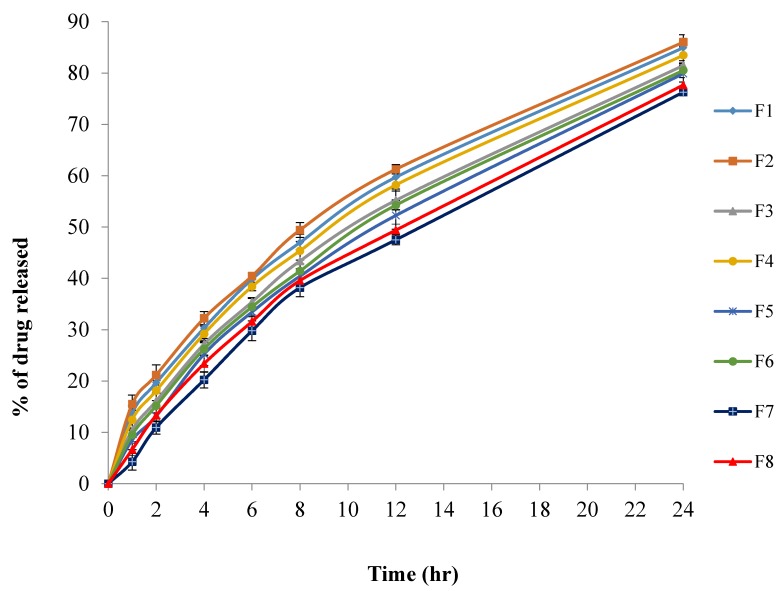
In vitro release profile of MIC from the prepared Transfersomes (F1–F8).

Figure 3 showed the in-vitro permeation profiles of MIC from the prepared Transfersomes. It was found that the permeation of MIC was done in two distinct phases. The initial phase represents rapid drug permeation and remained for about 8 h due to desorption of drug from the surface of Transfersomes, followed by a slow phase which stayed for at least 16 h due to the diffusion of drug through the lipid bilayer of the Transfersomes.

The prepared MIC Transfersomes were subjected to in vitro permeation to determine the transdermal flux, which was found to range from (93.48 ± 1.28 μg/cm^2^/h) for F1 to (105.42 ± 1.08 μg/cm^2^/h) for F6.

**Figure 4:**
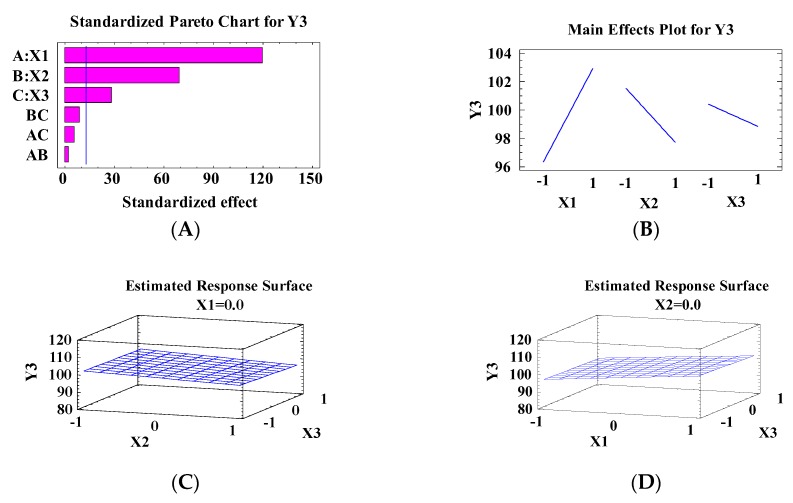

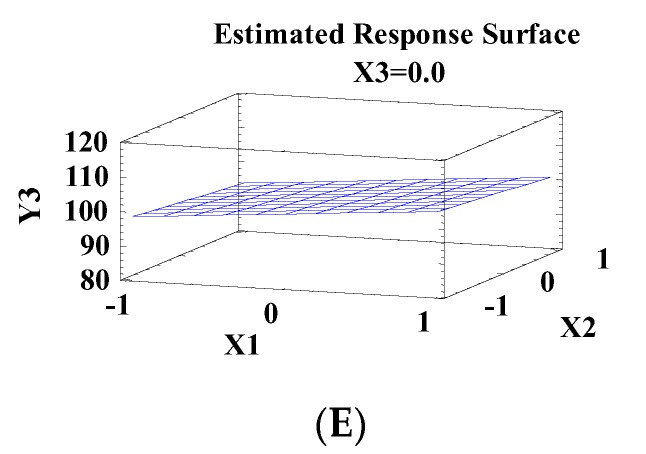
The effect of independent variables on the flux (Y3) including the Pareto chart (**A**), the main plot effect (**B**) and three-dimensional contour plots (**C**–**E**).

Figure 4A shows the standardized Pareto chart for the effect of the independent variables on Y3 (flux). It was found that X1, X2 and X3 have significant effects on Y3, with *p*-value <0.05, as investigated in Table 4. The linear regression models for flux of MIC through rat skin are represented in Equation (3), as obtained from multilevel factorial design study.

Y3 = 99.61 + 3.3 × X1 − 1.915 × X2 − 0.7825 × X3 + 0.07 × X1× X2 − 0.1625 × X1× X3 − 0.2525 × X2 × X3
(3)

The main effect plot, as shown in Figure 4B, represents the effect of independent variables in Y3. It was found that flux increased at high levels of X1 and low levels of X2 and X3, while decreasing at low levels of X1 and high levels of X2 and X3. These results also evidenced by estimated response surface, as shown in Figure 4C–E.

The permeation increased in the case of Tween 80 rather than Span 80, which could be attributed to an increase in the hydrophilicity in case of Tween 80, which causes it to act as a solubilizing agent for the drug, facilitating drug release [7].

The permeation rate also decreased as the total lipids increased, which may be due to the increased bilayer hydrophobicity and stability. Thus, the permeability decreased. When the phospholipid–surfactant ratio increased from 80:20 to 90:10, the release rate decreased. This might be attributed to the increase in the quantity of phospholipids causing an increase in the ratio which leads to a decrease in the permeability of the bilayer structure [3].

From the results of in vitro permeation and EE%, it was found that there was an inverse relationship between them. The higher the EE% of the MIC in prepared Transfersomes, the slower the in vitro permeation.

As shown in Table 5, the higher correlation coefficient values for Higuchi’s diffusion model suggested that the release of MIC from all prepared Transfersomes obeys Higuchi’s diffusion model.

### 3.5. Optimization of the Formulation Ingredients

**Figure 5:**
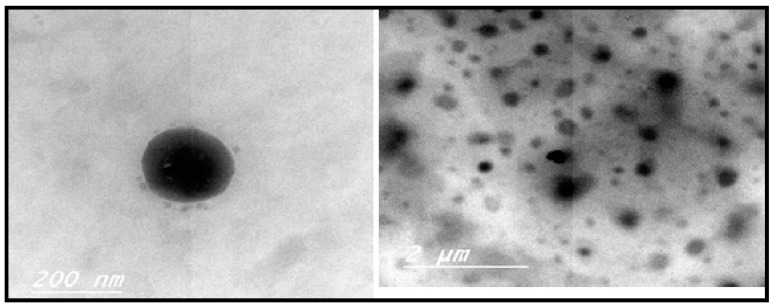
Transmission electron microscope image of the optimized MIC TRANSFEROSOME formulation (F5).

**Figure 6:**
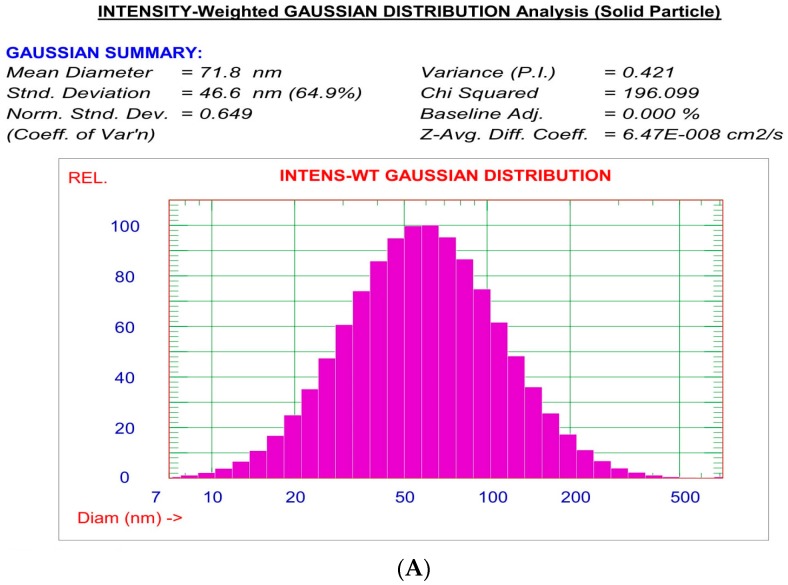

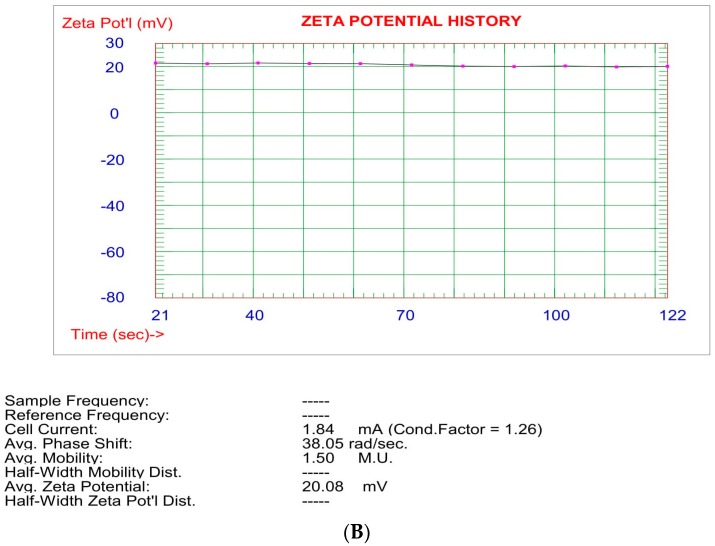
The particle size distribution curve (**A**) and zeta potential (**B**) of the optimized MIC TRANSFEROSOME formulation (F5).

Table 6 shows the composition of the optimized formula (F5). After optimization of formulation variables it was found that the optimized formulation was suggested to contain 80, 300 mg, and a ratio of (90:10) of X1, X2, and X3, respectively. As shown in Figure 5, TEM photographs of optimized MIC Transfersomes appeared as spherical, well identified, unilamellar nanovesicles. Figure 6 showed the particle size distribution and zeta potential of the optimized formula.

### 3.6. Fourier-Transform Infrared Spectroscopy (FTIR)

**Figure 7:**
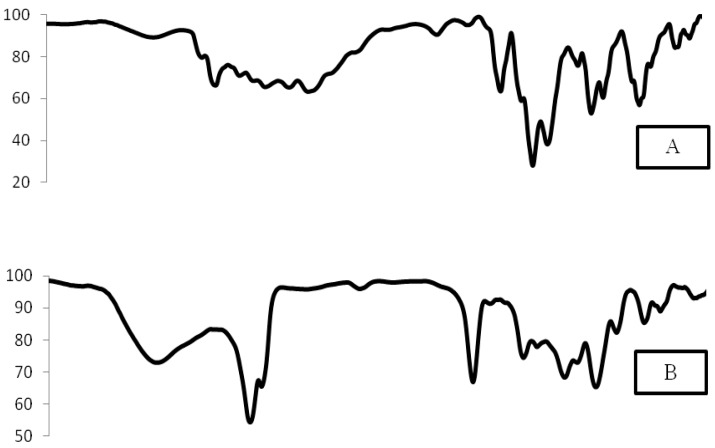

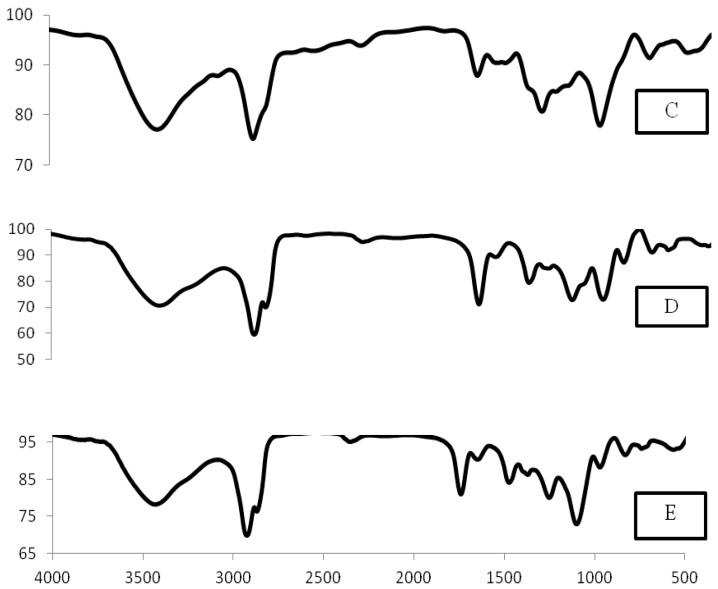
IR of (**A**) pure MIC, (**B**) physical mixture with Span 80, (**C**) physical mixture with Tween 80, (**D**) MIC Transfersomes with Span 80, and (**E**) MIC Transfersomes with Tween 80.

IR spectrophotometry has been employed as a useful tool to identify the drug excipient interaction. Figure 7 illustrates the IR spectrum of MIC, the physical mixture with Span 80, the physical mixture with Tween 80, MIC Transfersomes prepared with Span 80, and MIC Transfersomes prepared with Tween 80. The FTIR spectrum of MIC was characterized by bands at 3181 cm^−1^ (imidazole -C-N stretch), 3107 cm^−1^ (aromatic -C-H stretch), 2963 cm^−1^ (aliphatic -C-H stretch), 1547 cm^−1^ (aromatic -C-C), 1474 cm^−1^ (-CH_2_ bending), 1328 cm^−1^ (-C-N stretch), 1170 cm^−1^ (-C-O), 827 cm^−1^ (aromatic out-of-plane bend), and 637 cm^−1^ (-C-Cl) [1]. All the major peaks observed in the physical mixtures and MIC Transfersomes (prepared by either Span 80 or Tween 80) were similar to those recorded for MIC alone. The IR spectra indicate there were no interactions between the drug and excipient.

### 3.7. Differential Scanning Calorimetry (DSC)

**Figure 8:**
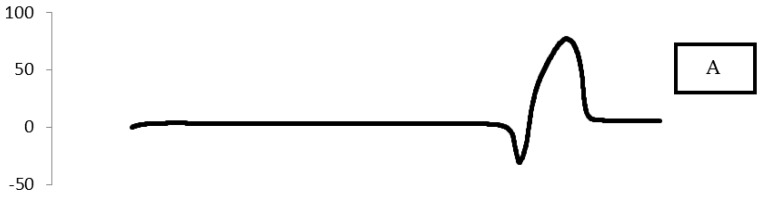

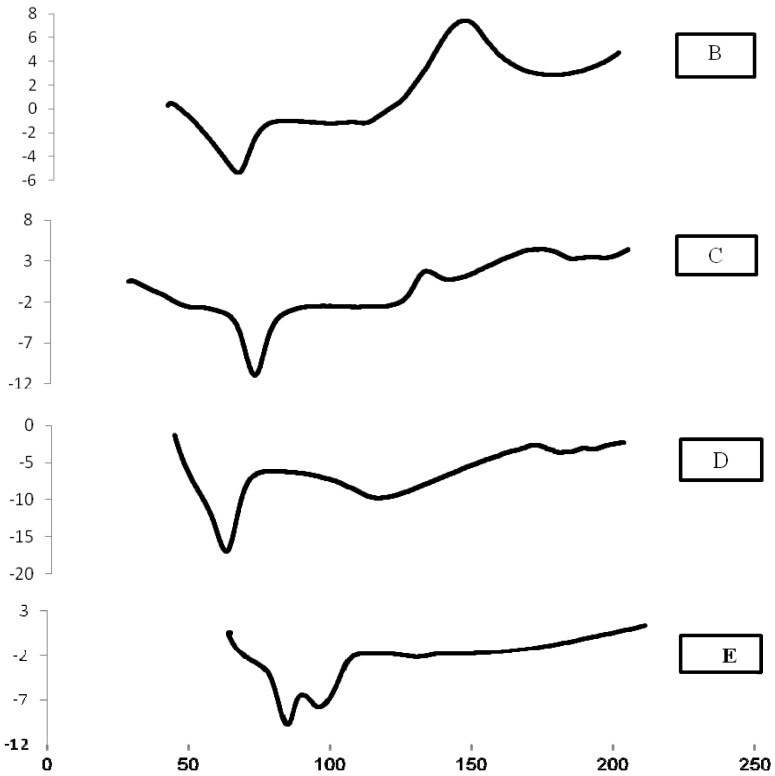
Differential scanning calorimetry (DSC) thermograms of (**A**) pure MIC, (**B**) a physical mixture with Span 80, (**C**) a physical mixture with Tween 80, (**D**) MIC Transfersomes with Span 80, and (**E**) MIC Transfersomes with Tween 80.

DSC is one of the most widely used calorimetric techniques to characterize the solubility and physical state of drug in lipid vesicles. The DSC analysis of the pure MIC, the physical mixture with Span 80, the physical mixture with Tween 80, MIC Transfersomes prepared with Span 80, and MIC Transfersomes prepared with Tween 80 were studied, as shown in Figure 8. The DSC analysis for pure MIC showed a large endothermic peak at 186.485 °C, which represents the melting point of MIC [1]. This peak disappeared in the DSC thermogram of MIC Transfersomes prepared with Span 80 and Tween 80. The disappearance of the melting endotherm of MIC suggested the presence of drug in a more soluble amorphous state [25].

The change in melting behavior of MIC could be due to the inhibition of its crystallization and solubilization in Transfersomes. Therefore, it could be concluded that the MIC in the prepared Transfersomes was in an amorphous form. The physical state transformation of a drug to an amorphous or partially amorphous state leads to a high-energy state and high disorder, resulting in enhanced solubility [24].

### 3.8. Formulation of an MIC Transfersomal Gel

An MIC transfersomal gel was prepared using 1% Carbapol 934 as a gelling agent. The concentration of MIC in the prepared transfersomal gel was 2% w/w, as in the Daktarin® cream 2%.

### 3.9. Evaluation of MIC Transfersomal gel and Daktarin® Cream 2%

Both the MIC transfersomal gel and Daktarin® cream 2% were smooth with a homogenous appearance. The spreadability values were 10.6 ± 0.73 and 7.2 ± 0.85 cm, respectively, which indicates that they can be spared easily on skin surface with little stress. The pH values were found to be 5.74 ± 0.38 for MIC transfersomal gel and 6.17 ± 0.94 for Daktarin® cream 2%, which were considered within the normal range of pH for topical preparations. The actual drug content of the MIC transfersomal gel was found to be 98.27 ± 1.4%, while for Daktarin® cream 2%, the content was found to be 95.93 ± 2.2% which represents good content uniformity. The viscosity of both MIC transfersomal gel and Daktarin® cream 2% was found to be 3.62 ± 0.74 and 5.54 ± 0.43 Pa/s, respectively (Table 7).

### 3.10. In Vitro Rat Skin Permeation of MIC from Transfersomal Gel and Daktarin® Cream (2%)

**Figure 9:**
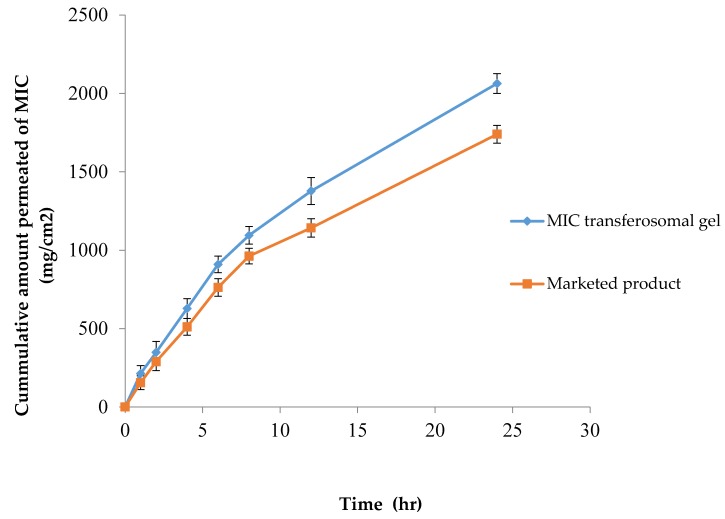
The cumulative amount of MIC which permeated through rat skin from the transfersomal gel in comparison with Daktarin® cream 2%.

The permeation profiles of MIC from prepared transfersomal gel and Daktarin® cream 2% are illustrated in Figure 9. It was found that the cumulative amount of MIC delivered from transfersomal gel was 2063.24 μg, which was significantly higher than the amount delivered by Daktarin® cream 2%, which was 1739.71 μg (*p* < 0.05). The improved permeation of MIC from MIC transfersomal gel may be attributed to high flexibility of Transfersomes, so they can penetrate the skin easily and overcome the barrier function by squeezing through the intracellular lipid of the stratum corneum [26]. Also, after application of Transfersomes on the skin, they move from the dry stratum corneum to a deep hydrated layer under the effect of the osmotic gradient, and the presence of surfactant in the structure of Transfersomes helps in solubilizing the lipid in the stratum corneum, permitting a high penetration of the vesicles [5].

As shown in Table 8, it is clear that there are high correlation coefficient values with the Higuchi diffusion model, suggesting that the permeation of MIC from the prepared transfersomal gel and Daktarin® cream 2% can be best described by Higuchi’s diffusion model [1].

### 3.11. Permeation Data Analysis

As shown in Table 9, the steady state flux was higher in the case of MIC transfersomal gel than in the case of the Daktarin® cream 2%. The steady state flux after 24 h for MIC transfersomal gel was 85.968 µg cm^−2^ h^−1^, while for Daktarin® cream 2% the value was 72.488 μg cm^−2^ h^−1^. It was found that there was a direct relationship between steady state flux and permeability coefficients, as represented in Table 9. The permeability coefficient of MIC transfersomal gel also was higher than (Daktarin® cream 2%). The previous results could be attributed to the high deformability and flexibility of Transfersomes, which allowed them to overcome skin barrier properties [1,7].

### 3.12. The In Vitro Antifungal Activity

**Figure 10:**
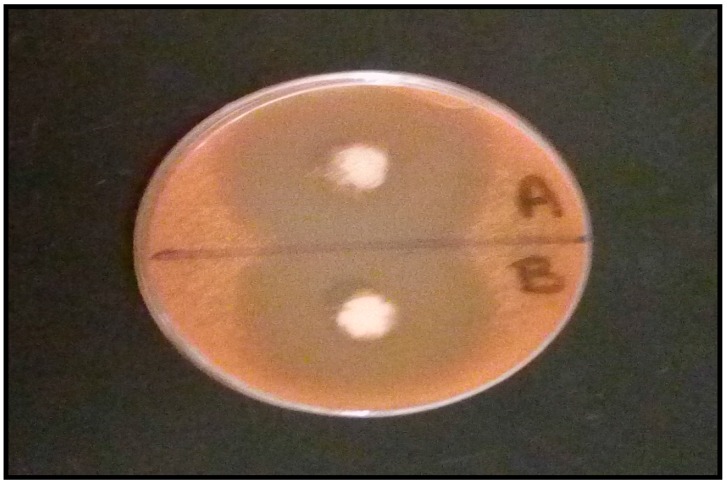
Antifungal activity test showing zone of inhibition for MIC transfersomal gel (**A**) and (Daktarin® cream 2%) (**B**).

The antifungal activity of the prepared MIC transfersomal gel against *Candida albicans* was evaluated in comparison to Daktarin® cream 2% through the cup plate method [1]. The antifungal activity was measured in terms of the diameter of the zone of inhibition [27]. Figure 10 showed the results of zone of inhibition; it was found that the antifungal activity area of the MIC transfersomal gel (55 mm) was greater than that of Daktarin® cream 2% (50 mm), which may be attributed to the high flexibility of Transfersomes, facilitating its penetration through the cell walls of *Candida albicans* fungi, and the inhibition of ergosterol biosynthesis, which results in fungal cell membrane lysis and cell death [7].

### 3.13. Treatment Effect of the MIC Transfersomal Gel

**Figure 11:**
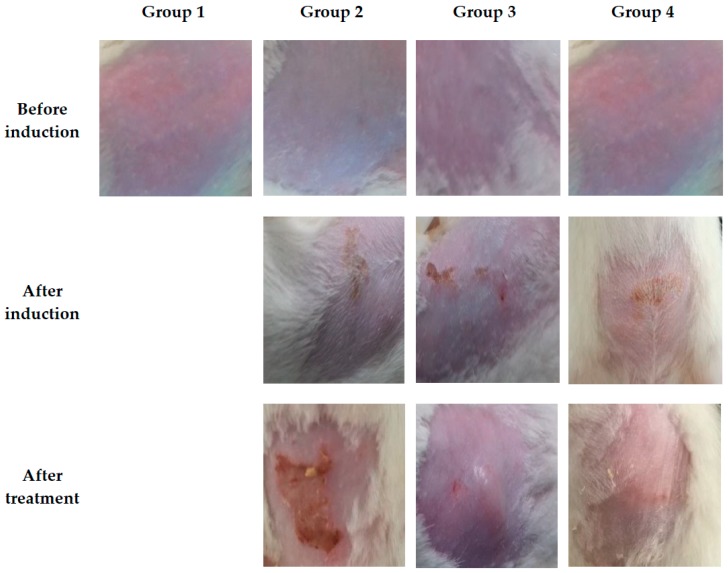
Skin samples of different rats before, after fungal infection using *Candida albicans* and after treatment.

Evaluation of antifungal activity using *Candida albicans* is widely used [28]. Before induction of cutaneous fungal infection, all animals showed normal skin structure without any clinical features of fungal infection, for example inflammation, edema, cracking, or color changes, as shown in Figure 11. After induction of fungal infection, the animals showed grayish or purple patches, inflammation, edema, and scaling and cracking of the skin. After treatment with Daktarin® cream 2%, the edema and other inflammation disappeared, while the scars were still present. On the other hand, the MIC transfersomal gel showed a normal skin with slight inflammation, as represented in Figure 11.

### 3.14. Histopathological Examination

**Figure 12:**
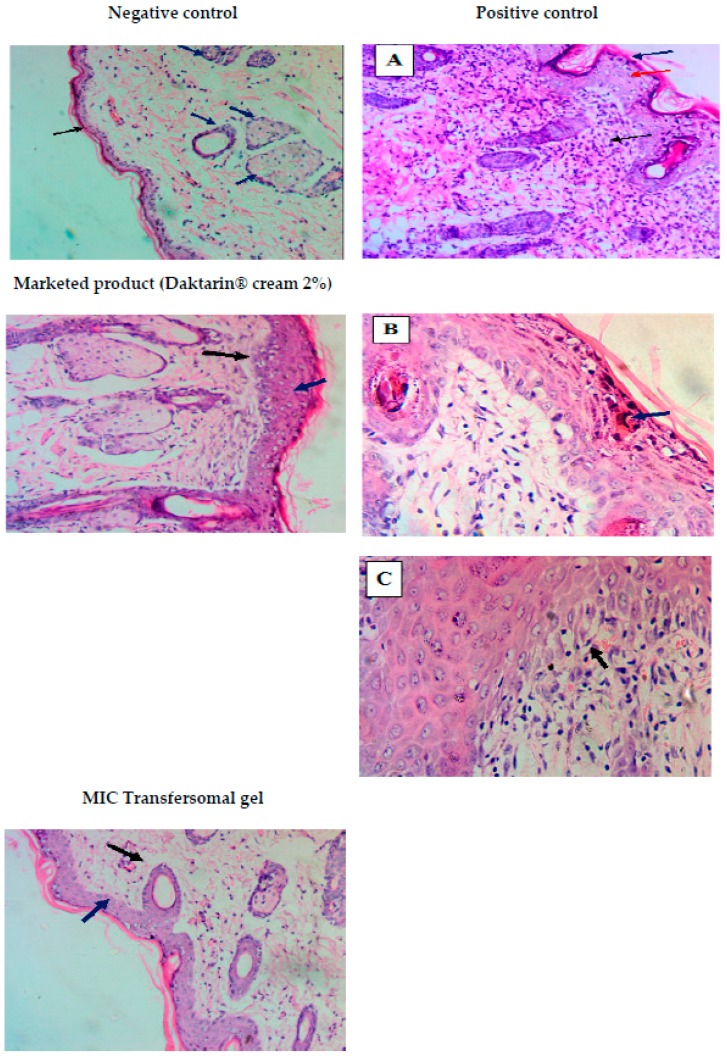
Treatment effect of MIC on the histopathhological changes in dermal fungal infection rats using *Candida albicans*: clinical postmortem examination histological images (H and E X 20 and 40) for the negative control, positive control, Daktarin® cream 2%, and MIC transfersomal gel.

As shown in Figure 12, normal animals showed a uniform dermis and epidermis (black and blue arrows) without any change in the normal structure of the skin. On the other hand, positive control group animals showed focal acanthosis (red arrow, A) with a mild compact hyperkeratosis layer (blue arrow, A). At the same time, fungal hyphae in the superficial epidermal layer was present (Blue arrow, B) and focal interface dermatitis (black arrow, C) was also seen, while the dermis layer showed dense, chronic inflammation (black arrow, A; see Figure 12). The animals which received the marketed product (Daktarin® cream 2%) showed an improvement in the dermis and epidermal layer, except for the focal acanthosis (blue arrow) and skin appendages (black arrow). On the other hand, the animals which received the MIC transfersomal gel showed a uniform skin structure in both the dermis and the epidermal layer, (see Figure 12). The previous results may be due to the deformability of Transfersomes which allows high permeability of MIC in the case of MIC transfersomal gel as compared to Daktarin® cream 2%, which contains free drug with a low ability for skin penetration [17]. The obtained results were supported by a quantitative evaluation of the pharmacological effect using the scoring system represented in Table 10.

## 4. Conclusions

From this study, it was concluded that the factorial design (2^3^) had the ability to obtain an optimized formula of miconazole nitrate, with high EE%, small particle size, and high transdermal flux. Also, the preparation of miconazole nitrate as transfersomal gel has the ability to overcome the barrier properties of the skin and increase the antifungal activity, as compared with the marketed product (Daktarin® cream 2%).

## Figures and Tables

**Table 1:** Formulation factors for the multilevel factorial design.

**Independent Factors**	**Low**	**High**
X1 = Type of surfactant	Span 80	Tween 80
X2 = Total lipids (mg)	300	600
X3 = Phospholipid:surfactant ratio (% w/w)	80:20	90:10
**Dependent Variables**	**Goal**	
Y1 = Entrapment efficiency (EE%)	Maximize	
Y2 = Vesicle size (nm)	Minimize	
Y3 = Flux (μg/cm^2^/h)	Maximize	

**Table 2:** The designed formulations of MIC Transfersomes.

Formulation No.	Drug (mg)	X1	X2	X3	Hydration Volume (mL)
F1	100	−1	1	1	10
F2	100	1	−1	1	10
F3	100	−1	−1	−1	10
F4	100	−1	1	−1	10
F5	100	−1	−1	1	10
F6	100	1	−1	−1	10
F7	100	1	1	1	10
F8	100	1	1	−1	10

MIC: miconazole nitrate; X1: type of surfactant; X2: total lipids (mg); X3: phospholipid–surfactant ratio.

**Table 3:** Characterization of prepared MIC Transfersomes.

Formula No.	EE (%) (Y1)	Vesicle Size (nm) (Y2)	Flux (μg/cm^2^/h) (Y3)	Zeta Potential (mv)	Polydispersity Index (PDI)
**F1**	91.47 ± 1.85	63.5 ±0.604	93.48 ± 1.28	+16.98	0.365
**F2**	71.34 ± 0.35	82.8 ±0.549	104.09 ± 0.87	+28.77	0.301
**F3**	81.92 ± 0.97	74.4 ±0.627	98.69 ± 0.95	+23.63	0.393
**F4**	88.39 ± 0.34	68.0 ±0.654	95.17 ± 1.13	+35.74	0.428
**F5**	86.17 ± 1.57	71.8 ±0.649	97.90 ± 0.75	+20.08	0.421
**F6**	67.98 ± 0.66	84.5 ±0.684	105.42 ± 1.08	+30.01	0.468
**F7**	78.62 ± 1.68	72.8 ±0.649	99.84 ± 0.99	+18.46	0.421
**F8**	75.78 ± 0.98	75.5 ±0.633	102.29 ± 1.18	+21.86	0.401

**Table 4:** ANOVA test for (Y1), (Y2), and (Y3) of the prepared MIC Transfersomes.

**Y1 (Entrapment Efficiency)**					
**Source**	**Sum of Squares**	**Degrees of Freedom (DF)**	**Mean Square**	**F-Ratio**	***p*-Value**
**A: (X1)**	367.612	1	367.612	6960.69	**0.0076**
**B: (X2)**	90.1153	1	90.1153	1706.33	**0.0154**
**C: (X3)**	22.8826	1	22.8826	433.28	**0.0306**
**AB**	1.36951	1	1.36951	25.93	0.1234
**AC**	0.159612	1	0.159612	3.02	0.3323
**BC**	0.357012	1	0.357012	6.76	0.2338
**Total error**	0.0528125	1	0.0528125	-		
**Total (correlation.)**	367.612	7	-			
**Y2 (Vesicle Size)**					
**Source**	**Sum of Squares**	**DF**	**Mean Square**	**F-Ratio**	***p*-Value**
**A: (X1)**	181.451	1	181.451	2962.47	**0.0117**
**B: (X2)**	143.651	1	143.651	2345.33	**0.0131**
**C: (X3)**	15.9613	1	15.9613	260.59	**0.0394**
**AB**	2.53125	1	2.53125	41.33	0.0982
**AC**	1.05125	1	1.05125	17.16	0.1508
**BC**	1.20125	1	1.20125	19.61	0.1414
**Total error**	0.06125	1	0.06125	-		
**Total (correlation.)**	345.909	7	-			
**Y3 (Flux)**					
**Source**	**Sum of Squares**	**DF**	**Mean Square**	**F-Ratio**	***p*-Value**
**A: (X1)**	87.12	1	87.12	14400.00	**0.0053**
**B: (X2)**	29.3378	1	29.3378	4849.22	**0.0091**
**C: (X3)**	4.89845	1	4.89845	809.66	**0.0224**
**AB**	0.0392	1	0.0392	6.48	0.2383
**AC**	0.21125	1	0.21125	34.92	0.1067
**BC**	0.51005	1	0.51005	84.31	0.0691
**Total error**	0.00605	1	0.00605	-		
**Total (correlation.)**	122.123	7	-			

Y1: R-squared = 99.9891%, R-squared (adjusted for d.f.) = 99.9234%, Standard Error of Est. = 0.22981, Mean absolute error = 0.08125, Durbin–Watson statistic = 1.0. Y2: R-squared = 99.9823%, R-squared (adjusted for d.f.) = 99.8761%, Standard Error of Est. = 0.247487, Mean absolute error = 0.0875, Durbin–Watson statistic = 1.0.Y3: R-squared = 99.995%, R-squared (adjusted for d.f.) = 99.9653%, Standard Error of Est. = 0.0777817, Mean absolute error = 0.0275, Durbin–Watson statistic = 1.0.

**Table 5:** The calculated correlation coefficients for the in-vitro release of MIC from Transfersomes employing different kinetic orders or systems.

Formula No.	Correlation Coefficient (r)					
	Zero	First	Second	Diffusion	H-C	B-L
**F1**	0.9764	−0.9990	0.9664	**0.9994**	0.9989	0.9907
**F2**	0.9753	−0.9989	0.9648	**0.9991**	0.9988	0.9919
**F3**	0.9792	−0.9992	0.9718	**0.9996**	0.9987	0.9882
**F4**	0.9768	−0.9993	0.9698	**0.9995**	0.9986	0.9904
**F5**	0.9815	−0.9986	0.9716	**0.9992**	0.9988	0.9846
**F6**	0.9806	−0.9992	0.9730	**0.9995**	0.9989	0.9872
**F7**	0.9813	−0.9984	0.9760	**0.9990**	0.9978	0.9814
**F8**	0.9820	−0.9984	0.9741	**0.9994**	0.9983	0.9830

**Table 6:** Composition of factors and response for the optimized MIC Transfersomes.

**Independent variables**	**Low**	**High**	**Optimum**
X1 = Type of surfactant	Span 80	Tween 80	Span 80
X2 = Total lipid (mg)	300	600	300
X3 = Phospholipid: surfactant ratio (% w/w)	80:20	90:10	90:10
**Response**	**Optimum**		
Y1	86.0888		
Y2	71.7125		
Y3	97.9275		

**Table 7:** Evaluation of MIC transfersomal gel in comparison to Daktarin® cream 2%.

Formulation	MIC transfersomal gel	Marketed product (Daktarin® cream 2%)
**Homogeneity**	Good	Good
**Spreadability (cm)**	10.6 ± 0.73	7.2 ± 0.85
**pH**	5.74 ± 0.38	6.17 ± 0.94
**Drug content (%)**	98.27 ± 1.4	95.93 ± 2.2
**Viscosity (Pa/s)**	3.62 ± 0.74	5.54 ± 0.43

**Table 8:** The calculated correlation coefficients for the in vitro permeation of MIC from transfersomal gel and the marketed product employing different kinetic orders or systems.

Formula Number	Correlation Coefficient (r)					
	Zero	First	Second	Diffusion	H-C	B-L
**MIC transfersomal gel**	0.9755	−0.9986	0.9914	0.9990	0.9944	0.9917
**Marketed product (Daktarin® cream 2%)**	0.9715	−0.9937	0.9977	0.9978	0.9882	0.9935

**Table 9:** Permeation parameters of MIC transfersomal gel in comparison to the marketed product (Daktarin® cream 2%).

Permeation Parameters	MIC Transfersomal Gel	Marketed Product (Daktarin® Cream 2%)
**Steady state flux (Jss; µg cm^−2^h^−1^)**	85.968 ± 0.45	72.488 ± 0.78
**Permeability coefficient (cm h^−1^)**	0.0172 ± 0.18	0.0145 ± 0.24

**Table 10:** Scoring system of histopathological examination of antifungal activity for MIC transfersomal gel compared to the marketed product.

Group	Fungal Hyphae	Acanthosis	Hyperkeratosis	Interface Dermatitis	Dermis	Inflammation
**Negative control**	0	0	0	0	0	0
**Positive control**	1	1	1	1	2	2
**Marketed product**	0	1	0	0	0	0
**MIC transfersomal gel**	0	0	0	0	0	0

Hyphae: 0 = Absent, 1 = Present; Acanthosis: 0 = absent, 1 = Focal, 2 = Diffuse; Hyperkeratosis: 0 = absent, 1 = Mild, 2 = Remarkable w/o parakeratosis; Interface dermatitis: 0 = absent, 1 = Mild, 2 = Remarkable; Dermis: 0 = Uniform, 1 = Mild chronic inflammation, 2 = Dense chronic inflammation.
